# Impact of an exercise program on acylcarnitines in obesity: a prospective controlled study

**DOI:** 10.1186/1550-2783-9-22

**Published:** 2012-05-10

**Authors:** René Rodríguez-Gutiérrez, Fernando J Lavalle-González, Laura E Martínez-Garza, Erick Landeros-Olvera, Juan C López-Alvarenga, Maria R Torres-Sepúlveda, Jose G González-González, Leonardo G Mancillas-Adame, Bertha Salazar-Gonzalez, Jesus Z Villarreal-Pérez

**Affiliations:** 1 Endocrinology Division, Internal Medicine Department, “Dr. José E. González”, University Hospital and Medical School of the Universidad Autónoma de Nuevo León, Ave. Madero y Ave. Gonzalitos s/n, Colonia Mitras Centro, Monterrey, Nuevo León, 64460, Mexico; 2 Genetics Department, Medical School of the Universidad Autónoma de Nuevo León, Ave. Madero y Dr. Eduardo Aguirre Pequeño s/n, Colonia Mitras Centro, Monterrey, Nuevo León, 64460, Mexico; 3 Cardiovascular Exercise Laboratory, Nursing School of the Benemérita Universidad Autónoma de Puebla, 4 sur 104, Centro Histórico, Puebla, 72000, Mexico; 4 Investigation Department of the Hospital General de Mexico, O.D, Dr. Balmis No.148, Col. Doctores, Delegación, Cuauhtémoc, 06726, Mexico; 5 Investigation Department, Nursing School, Universidad Autónoma de Nuevo León, Av. Gonzalitos 1500 Norte, Colonia Mitras Centro, Monterrey, Nuevo León, 64460, Mexico; 6 Servicio de Endocrinología, Hospital Universitario Dr. José E. Gonzalez, Ave. Madero y Gonzalitos s/n, Colonia Mitras Centro, Monterrey, Nuevo León, 64460, Mexico

**Keywords:** Beta-oxidation, Aerobic exercise, Diabetes mellitus, Overweight, Obesity, Lipotoxicity

## Abstract

**Background:**

Acylcarnitine (AC) transport dysfunction into the mitochondrial matrix is one of the pathophysiological mechanisms of type 2 diabetes mellitus (DM). The effect of an aerobic exercise (AE) program on this condition in obese subjects without DM is unclear.

**Methods:**

A prospective, randomized, longitudinal, interventional study in a University Research Center involved a 10-week AE program in 32 women without DM and a body mass index (BMI) greater than 27 kg/m^2^. (Cases n = 17; Controls n = 15). The primary objective was to evaluate the influence of a controlled AE program on beta-oxidation according to modifications in short, medium, and long-chain ACs. Secondary objectives were to define the behavior of amino acids, and the correlation between these modifications with metabolic and anthropometric markers.

**Results:**

The proportion of dropouts was 17% and 6% in controls and cases, respectively. In cases there was a significant reduction in total carnitine (30.40 [95% CI 28.2 to 35.6]) vs. (29.4 [CI 95% 25.1 to 31.7]) *p =* 0.0008 and long-chain AC C14 (0.06 [95% CI 0.05 to 0.08]) vs. (0.05 [95% CI 0.05 to 0.09]) *p =* 0.005 and in C18 (0.31 [95% CI 0.27 to 0.45]) vs. (0.28 [95% CI 0.22 to 0.32]) *p =* 0.03. Free fatty acid levels remained without change during the study in both groups.

**Conclusion:**

In conclusion, a controlled 10-week AE program improved beta-oxidation by reducing long-chain ACs. This finding highlights the importance that AE might have in avoiding or reverting lipotoxicity, and in consequence, improving insulin sensitivity and pancreatic beta cell functional reserve.

## Background

Diabetes Mellitus (DM) and obesity represent an annual cost of $132 and $147 billion dollars, respectively, for the United States Healthcare System [1-3]. Their incidence and severity have increased since the 1970s and it is estimated that by 2050 one third of the population in the United States will suffer from DM and half will be overweight or obese [4,5]. In Mexico, the problem is no less impressive since from 1988 to 2006 the prevalence of overweight and obesity went from 35% to 70% and the prevalence of DM in 2006 was almost 15% [6,7]. Obesity is one of the risk factors with the greatest impact on the development of DM and insulin resistance. The latter abnormality together with pancreatic beta cell dysfunction represent the initial pathophysiologic basis of type 2 DM [8,9]. Other important mechanisms have recently been identified, such as entero-insular axis dysfunction, increase in glucagon secretion, impaired renal reabsorption of glucose, brain insulin resistance, and lipotoxicity [10-16].

Impairment in long-chain acylcarnitine (AC) transfer to the mitochondrial matrix that results from dysfunction of carnitine palmitoyltransferase-1 (CPT1), leads to the accumulation of AC in cells [17,18]. This abnormality is one of the causes of lipotoxicity, which has been implicated as one of the mechanisms responsible for insulin resistance in liver and muscle, and of pancreatic beta cell dysfunction [19-21]. It is still debated whether this mitochondrial dysfunction is inherited or acquired and whether or not it is reversible. Recently, Mihalik *et al*. (*2010*), using tandem mass spectrometry in 10 type 2 DM and 14 obese patients, demonstrated the accumulation of plasmatic long-chain AC in the mitochondria and its relationship to insulin resistance after an overnight fast and four hours on an euglycemic clamp [22]. Studies with thiazolidinediones, on the other hand, have shown that these consequences of lipotoxicity can be prevented or reversed in subjects with type 2 DM [23,24]. Hiatt *et al*. (1989) demonstrated that a change occurred in the patterns of AC when they evaluated the influence of an episode of aerobic exercise (AE) of variable intensity in six healthy volunteers [25]. However, it is unknown whether this effect on the pattern of AC in a single episode of AE can be repeated or modified when carrying out a long term AE program. The influence of an AE program on the pattern of AC has not been studied in non-diabetic overweight or obese individuals. The identification of favorable changes in the ACs pattern of these populations, when subjected to an AE program, could be useful to modify the consequences of lipotoxicity.

We designed a randomized, prospective, longitudinal, experimental study in a group of obese or overweight individuals who underwent a 10-week AE program. Our main purpose was to define the influence of an AE program on beta-oxidation and fatty acid transport in mitochondria according to changes in total carnitine and short, medium, and long-chain ACs. We were also interested in studying the behavior of essential and nonessential amino acids, and analyzing the correlation of these changes with the determination of metabolic and anthropometric markers that can be modified with a controlled AE program.

## Subjects and methods

### Subjects

After obtaining approval from the Research and Ethics Committee and informed consent from each subject, we began the study. Participants were recruited through advertisements placed in different parts of the health campus of the Universidad Autónoma de Nuevo León. A total of 36 women, aged 18 to 24 years with a body mass index (BMI) greater than or equal to 27 kg/m^2^ were included. We excluded individuals who had exercised periodically in the last 3 months, subjects who had a weight change greater than 10% in the last 6 months or who were taking medications that alter insulin sensitivity, or lipid lowering or antihypertensive drugs during this period. We also excluded individuals with DM, hypertension, dyslipidemia or who smoked in the last 6 months.

### Study protocol

The participants were consecutively and randomly assigned to one of two groups: cases and controls. In order to prevent a change in calorie intake that would lead to a modification in body weight, which in turn would indirectly affect the effects of exercise, all participants received individual and group nutrition education. Before starting the AE program, all participants were measured for anthropometric indexes, such as weight, height, BMI, waist, hips, percentage of body fat and lean mass. Also, after a 12 hours overnight fast, blood samples were drawn for determination of AC, free fatty acids, amino acids, glucose, insulin, total cholesterol, triglycerides, low-density lipoprotein (LDL), high-density lipoprotein (HDL), leptin, adiponectin and tumor necrosis factor alpha (TNF alpha). These anthropometric measures and laboratory studies were performed at the beginning and at the end of the AE program.

The duration of the controlled AE program in both groups was 10 weeks. The control group received a manual with a gradual and progressive dose of exercise, based on recommendations of the American College of Sports Medicine, using the Borg scale for the perception of exercise intensity [26,27]. Exercise was performed as the subject wished; it was not controlled or supervised. The case group, on the other hand, received a controlled and supervised AE intervention during the same time period, with a frequency of five times a week and a duration of 20 minutes in the first two weeks, reaching 40 minutes by the fourth week; half of the session consisted of jogging on a treadmill and the other half of ergonomic bike pedaling. During the first three weeks the intensity was 40%-50% of the heart rate reserve (HRR), then, from the fourth to sixth weeks, the HRR was 50%-60%. The last 4 weeks were at a HRR of 60% to 80%.

### Measures

To perform exercise TRUE Z8 Soft-System treadmills and TRUE Z8 ergonomic bikes (TRUE Fitness Technology, Inc. St. Louis, MO) were used. The HRR was monitored with an Ekho Model E-15 heart rate monitor (Ekho Brand Americas, LLC, Minneapolis, MN). Calculation of the HRR to the percentage of desired intensity was performed in a personalized manner according to the Karvonen method (ACSM, 2010), using the following formula: HRR = ([maximum heart rate - resting heart rate] x desired percentage) + resting heart rate (26). AC and amino acids were analyzed in an API 2000 Triple Quadrupole Mass Spectrometer (PerkinElmer, Waltham, MA) coupled to a series 200 micropump and autosampler (PerkinElmer) using a Neogram kit for AC and amino acid spectrometry in tandem (PerkinElmer). Waist-hip circumference (WHC) and BMI measurements were performed according to recommendations of the National Institutes of Health [28]. BMI was calculated with the following formula: BMI = (weight in kg)/(height in m²). Weight and height were determined on a Seca 700 calibrated mechanical scale with a stadiometer (TAQ, Sistemas Médicos, Mexico City, Mexico). Anthropometric measurements were performed by an ISAK (International Society for the Advance of Kinanthropometry) certified individual who was blinded to participant´s information.

The percentage of body fat and lean body mass were determined using air displacement plethysmography (BodPod, Life Measurement, Inc., Concord, CA). The XY multiplex platform (Luminex®, Luminex Corporation Austin, TX) with calibration microspheres was used with MagPlex software that reads magnetic strips for reporting plasma concentrations of adiponectin, TNFα and leptin. Plasma glucose measurement was performed using the glucose oxidase method (Adiva 1650 Chemistry system, Bayer, Leverkueusen, Germany; intraassay CV <2%); insulin was measured using an immunoassay electrochemiluminescence kit (Roche Diagnostics Indianapolis, IN; intraassay CV <2%), lipid profile was determined with an Immulite 2000 analyzer (Diagnostic Products Corporation, Los Angeles, CA; CV <8% for all measurements). HOMA-IR was calculated using the following formula: HOMA-IR = fasting serum insulin (uU/ml) x fasting plasma glucose (mmol/ml)/22.5 [29]. A HOMA less than or equal to 2.5 was considered the normal cutoff value because a higher value has been associated with increased cardiovascular risk in Mexican-American population [30].

### Statistical analysis

All results are presented as medians and 95% confidence intervals (CI), unless otherwise stated. Differences were considered statistically significant if *P* was equal or less than 0.05. To evaluate the anthropometric variables of age and height we used Student´s *t* test. For the rest of the anthropometric, biochemical, AC and amino acid variables nonparametric tests were used: the Mann–Whitney U for comparison of different groups and the Wilcoxon rank test for comparison of values within a group. Sample size was calculated based on a change in adiponectin through the AE intervention, with a power of 80%, an effect size of 38% and a significance level of 0.05. This resulted in an n per group of 16 subjects. Statistical analysis was performed with SPSS Statistics 15.0 (SPSS Inc., Armonk, NY) and with MedCalc Version 11.4.4.0 for Windows (MedCalc Software, Ghent, Belgium).

## Results

### Study population

Eighteen participants were randomized into each group. In the control group 15 out of 18 participants (83%) completed the study period, in contrast to 17 out of 18 (94%) in the case group. The four participants who dropped out of the study did so within the first 2 weeks. In the control group 13 out of 15 participants attended at least 3 of the 5 uncontrolled weekly workout sessions throughout the study, whereas in the case group, of the 17 participants, 100% attended at least 4 weekly controlled AE sessions and 14 attended all sessions. The mean age of the case group and controls was 20.3 years ± 1.44 SD and 21.5 years ± 2.19 SD, respectively (p = 0.08).

### Anthropometric and metabolic variables

#### A: Baseline characteristics

The baseline anthropometric and metabolic characteristics of each group are shown in Table 1. Initially there were 8 vs. 9 participants overweight in the case group and controls, respectively. There were 9 vs. 6 cases and controls, respectively, with obesity (*p* = 0.23). There was also no statistically significant differences between case and control groups when the median of all anthropometric measures including weight, height, BMI, percent body fat, lean body mass, waist, hips and waist/hip ratio were evaluated. Although the median weight in the group of cases vs. controls was higher (81.10 kg [95% CI 72.08 to 84.70]) vs. (72.20 kg [95% CI 66.55 to 80.75]) *p* = 0.06, this was not significant. This discrepancy was attenuated when BMI was compared between the groups of cases and controls (30.50 kg/m^2^ [95% CI 28.50 to 32.69]) vs. (28.89 kg/m^2^ [95% CI 27.78 to 31.12]) p = 0.15, respectively. The metabolic variables were not statistically different in levels of adiponectin, leptin, insulin, TNF alpha, total cholesterol, triglycerides, HDL, LDL, and levels of free fatty acids. There was only a significant difference in median fasting glucose, 74 mg/dl (95% CI 73 to 78.96) in the case group vs. 84 mg/dl (80.26 to 88.0) in the control group (p = 0.003); while the median HOMA was 2.2 (95% CI 1.6 to 3.0) vs. 2.9 (CI 95% 2.3 to 5.2) for cases and controls, respectively (p = 0.047).

**Table 1:** Baseline and End of Study Anthropometric and Metabolic Measures in Controls and Cases

	**Baseline**		***P*+**	**End of the Study**		***P*+**	**A vs. C‡**	**B vs. D‡**
	**Control (A) n = 15**	**Case (B) n = 17**		**Control (C) n = 15**	**Case (D) n = 17**			
Age, y §	21.5 ± 2.19	20.3 ± 1.44	0.08	72.40 (65.66 – 82.08)	76.30 (69.90 – 82.22)	0.39	0.80	0.0003*
Weight, kg.	72.20 (66.55 – 80.75)	81.10 (72.08–84.7)	0.06					
Height, cm	157 (151.26 – 163.47)	161 (156 - 164)	0.24					
BMI kg/m^2^	28.89 (27.78 – 31.12)	30.50 (28.50 – 32. 69)	0.15	29.10 (27.73. 30.88)	28.80 (27.50 – 30.78)	0.74	0.76	0.0002*
FM kg	26.7 (23.15 – 31.26)	32.6 (23.51 – 34.4)	0.08	27.60 (23.50 – 31.01)	29.40 (23.12 – 33.07)	0.67	0.58	0.0005*
FFM kg	45.70 (42.13 – 48.26)	48.70 (46.20 – 50.29)	0.08	44.80 (41.75 – 47.94)	47.90 (45.80 – 49.39)	0.06	0.13	0.03*
Waist cm	83 (80.38 – 88)	86.40 (82.02 – 91.98)	0.24	82.50 (79.76 – 86.15)	83 (79.50 – 86)	0.74	0.11	< 0.0001*
Hip cm	108 (102.26 – 110.62)	112.5 (105.04 – 115.46)	0.07	106.5 (102.52 – 108.73)	108 (103 – 111)	0.76	0.54	0.0002*
Waist to Hip Ratio	0.79 (0.76 - 0.81)	0.78 (0.77 - 0.81)	0.80	0.77 (0.75 - 0.80)	0.78 (0.75 - 0.79)	0.63	0.27	0.04*
Adiponectin ug/ml	11.54 (7.88 – 15.26)	11.72 (7.29 – 15.06)	0.61	12.33 (8.36 – 15.60)	15.76 (9.96 – 23.44)	0.32	0.80	< 0.0001*
Leptin ng/ml	30.33 (25.30 – 36.06)	28.31 (23.82 – 35.12)	0.71	29.42 (21.51 – 37)	18.13 (12.94 – 24.31)	0.002*	0.45	0.03*
TNFa pg/ml	4.44 (4.10 – 6.14)	4.33 (2.90 – 5.31)	0.25	5.05 (4.12 – 6.76)	4.10 (3.53 – 4.98)	0.036*	0.12	0.93
Insulin, mg/dl	13.72 (11.47 – 24.95)	12.01 (8.64–16.74)	0.14	12.73 (10.70 – 19.43)	12.89 (6.42 – 14.37)	0.12	0.01*	0.17
Glucose, mg/dl	84 (80.26 – 88)	74 (73–78.96)	0.003*	86 (82.26 – 87)	82 (76.01 – 87)	0.39	0.80	0.05*
CHOL, mg/dl	78 (59.05 – 149.02)	78 (62.03 – 111.79)	0.69	78 (65.79 – 113)	66 (59.03 – 99-95)	0.15	0.59	0.33
TGL mg/dl	160 (144.52 – 182.41)	153 (144.04 – 186.98)	0.87	165 (149.70 – 186.73)	168 (152.01 – 184. 91)	0.79	0.42	0.12
HDL mg/dl	49 (36.26 – 54.94)	45 (41.01 – 71.74)	0.39	45 (39.26 – 53.47)	44 (40 – 55.93)	0.98	0.84	0.32
LDL mg/dl	104 (98–109.47)	105 (102.01 – 109.98)	0.50	95 (90 – 102.41)	92 (80.12 – 97.98)	0.36	0.20	0.0001*
HOMA	2.88 (2.29 – 5.20)	2.22 (1.59 – 3.01)	0.047*	2.58 (2.29 – 4.20)	2.29 (1.47 – 2.83)	0.15	0.06	0.40
FFA mEq/L	0.40 (0.30 - 0.50)	0.40 (0.30 - 0.59)	0.39	0.50 (0.32 - 0.60)	0.40 (0.40 - 0.60)	0.86	0.27	0.23

^ Results are reported in median and 95% Confidence Interval, except age (§), which is mean ± SD.

FFA denotes Free-fat Mass, FM fat mass, BMI body mass index, W/H Waist to Hip Ratio, TNFa tumor necrosis factor alpha, CHOL Cholesterol, TGL Triglycerides, HOMA Homeostatic Model Assessment, HDL high-density lipoprotein, LDL low-density lipoprotein, FFA Free fatty acids.

+p Values where calculated by Mann–Whitney Test.

‡*p* Values where calculated by Wilcoxon Rank Test.

* Significant Result p < 0.05.

#### B: Comparison of baseline vs. End of the study in each group

In the control group there was no statistically significant difference in the anthropometric characteristics when compared before vs. after 10 weeks of the AE program (Table 1). However, in the case group after 10 weeks of an AE program a favorable change in all variables occurred, reaching statistical significance. It is noteworthy that in this group there was a decrease in the median weight of about 5 kg, 81.10 kg. (95% CI 72.08 to 84.69) vs. 76.30 kg (95% CI 69.90 to 82.22).

When baseline metabolic vs. endpoint variables were compared in the control group, insulin was the only variable with a statistically significant reduction, 13.72 uUI/ml (95% CI 11.47 to 24.95) vs. 12.73 uUI/ml (95% CI 10.70 to 19.43), p = 0.01. On the other hand, expected significant changes occurred in cases in leptin, adiponectin, and low-density lipoprotein levels. The mean plasma glucose, increased in a significant level, 74 mg/dl [95% CI (73.0 to 78.9) vs. 82 mg/dl (95% CI 76.01 to 87.6), p = 0.05. However, in this group plasma insulin levels remained unchanged.

#### C: Comparison between groups at the end of the study

After 10 weeks of AE, when contrasting the anthropometric variables among the groups there was no significant difference in any of the studied variables (Table 1). However, when the metabolic variables were compared, significantly lower values in the case group in leptin and TNF alpha were found.

### Acylcarnitines

At baseline, a difference in short-chain AC levels (C3DC and C4) between cases and controls was found; these were significantly higher in the control group (See Table 2). Also, the levels of a single medium-chain AC, C8, were significantly higher in controls. There were no differences between long-chain AC groups. At the end of the exercise program in the control group, a comparison of baseline vs. end AC levels showed a significant increase in short-chain AC C3 (0.65 [95% CI 0.54 to 0.82] vs. 0.77 [95% CI 0.64 to 0.93]) and long-chain AC C16OH (0.04 [95% CI 0.02 to 0.05] vs. 0.07 [0.04 to 0.09]). In the case group there was a significant decrease in total carnitine (30.40 [95% CI 28.21to 35.58] vs. 29.40 [95% CI 25.12 to 31.69]) and long-chain AC C14 (0.09 [95% CI 0.05 to 0.12] vs. 0.05 [95% CI 0.04 to 0.09]) and AC C18 (0.31 [95% CI 0.27-0.45] vs. 0.28 [95% CI 0.22 to 0.32]). In this group there was a significant increase in medium-chain AC C8 (0.06 [95% CI 0.04 to 0.07] vs. 0.10 [95% CI 0.07 to 0.12]), p = 0.03 and AC C5 (0.12 [95% CI 0.10 to 0.15] vs. 0.19 [95% CI 0.15 to 0.24]), p = 0.05. The comparison between groups at the end of the program showed a significant increase in medium-chain AC C10 (0.06 [95% CI 0.04 to 0.15] vs. 0.11 [95% CI 0.08 to 0.15]) in the case group only. See Table 2.

**Table 2:** Baseline and End of Study Acylcarnitines in Controls and Cases

	**Baseline**		***p*+**	**End of the Study**		***p*+**	***A vsC*‡**	***B vsD*‡**
	**Control (A) n = 15**	**Case (B) n = 17**		**Control (C) n = 15**	**Case (D) n = 17**			
C0	30.20 (24.80–34.31)	30.40 (28.21–35.58)	0.42	30.10 (24.23–34.74)	29.40 (25.12–31.69)	0.61	0.20	0.0008*
C2	8.23 (6.02–9.94)	7.21 (5.61–11.98)	0.94	6.78 (5.77–9.79)	6.89 (5.47–10.29)	0.95	0.22	0.24
C3	0.65 (0.54–0.82)	0.61 (0.49–0.74)	0.60	0.77 (0.64–0.93)	0.68 (0.50–0.84)	0.18	0.006*	0.35
C3DC	0.08 (0.07–0.10)	0.06 (0.04–0.08)	0.01*	0.08 (0.05–0.09)	0.06 (0.04–0.11)	0.89	0.38	0.32
C4	0.19 (0.14–0.20)	0.11 (0.07–0.16)	0.02*	0.18 (0.12–0.24)	0.13 (0.10–0.16)	0.10	0.27	0.48
C4DC	0.41 (0.25–0.56)	0.45 (0.33–0.53)	0.68	0.41 (0.30–0.53)	0.50 (0.33–0.54)	0.71	0.27	0.74
C5	0.14 (0.12–0.18)	0.12 (0.10–0.15)	0.77	0.16 (0.14–0.20)	0.19 (0.15–0.24)	0.06	0.63	0.050*
C5OH	0.20 (0.13–0.29)	0.25 (0.18–0.28)	0.48	0.22 (0.14–0.24)	0.24 (0.18–0.27)	0.29	0.59	0.96
C5:1	0.03 (0.02–0.4)	0.03 (0.02–0.5)	0.89	0.03 (0.02–0.06)	0.03 (0.02–0.05)	1.00	0.90	0.78
C5DC	0.09 (0.04–0.19)	0.09 (0.05–0.12)	0.40	0.08 (0.06–0.10)	0.08 (0.06–0.10)	0.18	0.48	0.14
C6	0.07 (0.04–0.09)	0.05 (0.04–0.08)	0.79	0.04 (0.03–0.08)	0.05 (0.03–0.07)	0.74	0.20	0.82
C6DC	0.07 (0.04–0.10)	0.06 (0.05–0.08)	0.25	0.06 (0.03–0.08)	0.06 (0.03–0.07)	0.82	0.22	0.78
C8	0.11 (0.07–0.14)	0.06 (0.04–0.07)	0.006*	0.09 (0.07–0.12)	0.10 (0.07–0.12)	0.79	0.20	0.039*
C10	0.07 (0.05–0.10)	0.07 (0.04–0.12)	0.71	0.06 (0.01–0.10)	0.05 (0.02–0.09)	0.04*	0.65	0.09
C10:1	0.09 (0.06–0.13)	0.08 (0.05–0.10)	0.34	0.07 (0.03–0.11)	0.08 (0.07–0.13)	0.41	0.15	0.61
C10:2	0.06 (0.01–0.10)	0.05 (0.02–0.09)	0.74	0.05 (0.03–0.10)	0.07 (0.03–0.10)	0.86	0.71	0.15
C12	0.07 (0.04–0.11)	0.07 (0.05–0.09)	0.66	0.07 (0.04–0.14)	0.08 (0.05–0.09)	0.61	0.38	0.30
C14	0.06 (0.04–0.09)	0.06 (0.05–0.08)	0.69	0.06 (0.04–0.10)	0.05 (0.05–0.09)	0.49	0.30	0.005*
C14:1	0.07 (0.02–0.10)	0.06 (0.05–0.08)	0.55	0.06 (0.05–0.09)	0.05 (0.04–0.10)	0.67	0.89	0.78
C14:2	0.03 (0.03–0.06)	0.04 (0.02–0.07)	0.49	0.05 (0.03–0.07)	0.03 (0.02–0.05)	0.12	0.30	0.17
C16	0.67 (0.52–0.67)	0.60 (0.50–0.73)	0.47	0.57 (0.45–0.68)	0.59 (0.50–0.68)	0.79	0.27	0.57
C160H	0.04 (0.02–0.05)	0.03 (0.03–0.05)	0.58	0.07 (0.04–0.09)	0.04 (0.02–0.05)	0.74	0.04*	0.37
C16:1	0.07 (0.06–0.10)	0.06 (0.03–0.08)	0.10	0.06 (0.05–0.07)	0.05 (0.04–0.07)	0.79	0.06	0.99
C16:1 OH	0.08 (0.06–0.09)	0.09 (0.07–0.11)	0.26	0.07 (0.04–0.09)	0.07 (0.05–0.10)	0.49	0.42	0.35
C18	0.37 (0.32–0.41)	0.31 (0.27–0.45)	0.24	0.36 (0.28–0.45)	0.28 (0.22–0.32)	0.27	0.80	0.03*
C18 OH	0.06 (0.03–0.10)	0.04 (0.03–0.08)	0.66	0.07 (0.03–0.11)	0.05 (0.03–0.11)	0.86	0.38	0.48
C18:1	0.64 (0.59–0.81)	0.74 (0.68–0.84)	0.13	0.64 (0.53–0.79)	0.73 (0.61–0.83)	0.24	0.76	0.92
C18:1 OH	0.03 (0.02–0.03)	0.02 (0.02–0.03)	0.42	0.02 (0.02–0.03)	0.02 (0.02–0.03)	0.95	0.84	0.43
C18:2	0.22 (0.18–0.33)	0.28 (0.22–0.32)	0.36	0.24 (0.21–0.28)	0.22 (0.17–0.30)	0.31	0.97	0.12

^All values are in μmol/l. Results are reported in Median and Confidence Interval 95%.

+p Values were calculated by Mann–Whitney Test.

‡*p* Values were calculated by Wilcoxon Rank Test.

* Significant Result p < 0.05.

### Amino acids

There was no difference found when the levels of amino acids between the groups at the beginning of the AE program were compared (Table 3). At the end of the exercise program a decrease in the levels of tyrosine and ornithine in the group of cases with respect to baseline was observed. In the control group there was no significant change when compared with their baseline. Finally, when comparing the final values between the groups there was only a significant decrease in tyrosine levels in the group of cases.

**Table 3:** Baseline and End of Study Amino Acids in Controls and Cases

	**Baseline**		***p*+**	**End of the Study**		***p*+**	***A vsC*‡**	***B vsD*‡**
	**Control (A) n = 15**	**Cases (B) n = 17**		**Control (C) n = 15**	**Case (D) n = 17**			
Alanine	213.00 (190.27 – 282.78)	238.00 (202.03 – 259.95)	0.59	240.00 (185.52 – 271.17)	208.00 (198.01 – 234.00)	0.59	0.84	0.09
Arginine	46.90 (40.51 – 62.78)	46.70 (38.55 – 52.69)	0.50	51.50 (32.61 – 68.11)	49.60 (37.35 – 59.99)	0.80	0.84	0.37
Citrulline	18.10 (14.95 – 20.41)	15.40 (14.20 – 15.99)	0.15	16.00 (12.96 – 18.42)	14.30 (12.61 – 17.18)	0.38	0.07	0.27
Glycine	200.00 (188.53 – 243.23)	224 (184.30 281.66)	0.42	205.00 (184.78 – 224.29)	208.00 (298.03 – 245.96)	0.34	0.89	0.40
Leucine	101.00 (84.59 – 108.20)	95.50 (85.85 - 101.97)	0.53	96.80 (89.02 – 111.67)	95.60 (91.83 – 104.93)	0.74	0.63	0.78
Methionine	42.90 (36.81 – 45.96)	40.10 (36.15 – 44.36)	0.50	44.00 (34.53 – 48.14)	40.20 (30.41 – 44.89)	0.23	0.76	0.54
Ornithine	74.20 (66.33 – 81.85)	79.40 (75.70 – 84.46)	0.28	69.20 (60.00 – 72.21)	66.00 (59.23 – 70.15)	0.40	0.21	0.003*
Phenylalanine	51.80 (44.61 – 53.71)	44.60 (43.20 – 49.09)	0.21	44.40 (40.06 – 49.91)	44.60 (42.90 – 47.67)	0.80	0.18	0.76
Tyrosine	49.80 (44.87 – 62.62)	45.50 (41.90 – 50.58)	0.26	45.90 (39.97 – 51.14)	41.50 (37.60 – 44.97)	0.05	0.16	0.05*
Valine	123.00 (97.69 – 153.35)	115.00 (101.09 – 142.67)	0.71	121.00 (102.11 – 141.35)	111.00 (98.99 – 124.87)	0.27	0.56	0.30

^ All values are in μmol/l. Results are reported in Median and 95% Confidence Interval.

+p Values were calculated by Mann–Whitney Test.

‡*p* Values were calculated by Wilcoxon Rank Test.

* Significant Result p < 0.05.

## Discussion

Our study showed that a 10-week AE program in a young population of obese women without DM significantly decreased levels of long-chain AC (C18 and C14), only in the case group, with a non-significant difference on plasma free fatty acids during the study period. Although there are many aspects that are still needed to be identified between the link of lipotoxicity and insulin resistance, it is well known that an increase in intracellular lipid levels leads to a decrease in insulin action [8,16,31]. If this is secondary to an excess of plasma free fatty acids and/or a decrease in their beta-oxidation is unclear [32]. This last defect in patients with type 2 DM and obesity has been shown to persist in the fasting state and is not removed after an insulin stimulus with a euglycemic clamp [33,34]. This disorder, also known as metabolic inflexibility, has been attributed to inhibition of CPT1 by malonyl-CoA leading to an inability to transport long-chain AC into the mitochondrial matrix and thus the dysfunction in beta-oxidation [21]. In our study, the identification of similar levels of free fatty acids at baseline as well as at the end of the intervention, suggests that beta-oxidation was improved, being partially reversed, likely due to an increase in CPT1 function, since a decrease in long-chain AC (C14 and C18) occurred only in the case group as a result of the AE program. This conclusion is strengthened by the fact that pairs of long chain ACs (C14 and C18) were those that were modified; the ACs pairs of up to 20 carbons accumulate in response to deterioration in beta-oxidation of fatty acids in contrast with the accumulation of odd ACs that result from the catabolism of amino acids, except for C4, which is derived from both processes [22]. It is important to point out that the baseline AC pattern was similar in both groups and agrees with that reported previously [22].

When interpreting the mechanism of decline in long-chain AC in the group of cases at the end of the study, it is necessary to analyze the influence of a change in caloric intake and a resulting decrease in body weight. The influence of these on beta-oxidation has also been an area of controversy [35,36]. In our study, both groups of participants were carefully instructed not to alter their caloric intake throughout the 10-week study. Consequently, any changes in body weight should be a consequence of the exercise program. Only the case group showed a significant weight loss at the end of the exercise program, which should be attributed to their better adherence and intensity to the AE program. In accordance with this concept is the fact that free fatty acid levels remained unchanged in both groups during the study. The favorable change in body weight and anthropometry only due to weight loss without exercise should not be regarded as the critical mechanism of metabolic flexibility recovery. Goodpasture *et al*. (*2003*), studying a group of 25 obese subjects without diabetes, found that restoration of metabolic flexibility may be obtained by a moderate weight loss as a consequence of an AE program but not when compared to an equivalent weight loss obtained from a program based solely on calorie restriction [34]. Consistent with this, in our study, only the case group had a decrease in long-chain AC as a result of improved beta-oxidation. A critical factor that strengths the AE program in the case group, was that all the anthropometric and metabolic variables where modified according to what is already well known [37-39]. As well, amino acids, ornithine and tyrosine decreased as previously described by AE [40].

Another important finding in our study was that in the case group medium-chain AC C8 and C5 increased at the end of the exercise program. Unlike long-chain AC, medium chain AC did not depend on CPT1 for transfer to the mitochondrial matrix. This would reinforce the theory that improvement in beta-oxidation occurs mainly as a result of an increase in CPT1 activity. Recent studies agree with this finding, suggesting that intermediate products such as beta-oxidation of medium-chain AC accumulate in patients with type 2 DM, reflecting that a more complex beta-oxidation defect may be present; this abnormality was not reversed by the AE program our participants underwent [31,35,41]. It could be that a more intense AE program, with a greater length of time, in an older population and with insulin resistance could improve this defect in beta-oxidation in subjects who are obese or have diabetes.

If the mitochondrial capacity of beta-oxidation is a permanent or reversible defect is a matter of controversy. Recent studies have found that mitochondrial beta-oxidation is reduced in patients with type 2 DM and that this abnormality is reversible [42,43]. In a group of 10 patients with obesity and type 2 DM, Toledo *et al*. (*2007*), in skeletal muscle biopsies, showed an improvement in beta-oxidation after a moderate 16-week AE program. In another study in 21 obese subjects undergoing a 16-week AE program, muscle biopsies at the end of the study identified an increased number of mitochondria and an increased amount of lipid droplets consistent with the beneficial metabolic effects. Our results show that a controlled 10-week AE program was able to improve, in the case group, beta-oxidation.

## Conclusions

A 10-week AE program led to well known anthropometric and biochemical modifications in a young group of obese women without DM, improved beta-oxidation by decreasing long-chain ACs probably due to an increase in CPT1 function, being this a consequence of the physical activity and the weight loss that occurred as a direct result of the AE program. These findings warrant longer-term studies to analyze their effects on long and medium-chain AC and the permanence of these modifications after stopping exercise. So far our results suggest that a long term AE program might likely improve lipotoxicity and, consequently, insulin action and pancreatic beta cell functional reserve.

## Abbreviations

DM: Diabetes mellitus; ACs: Acylcarnitines; AE: Aerobic exercise; CPT1: Carnitine palmitoyl transferase-1; BMI: Body mass index; HRR: Heart rate reserve; MS/MS: Tandem mass spectrometry.

## Competing interests

The authors declare they have no competing interests.

## Author’s contributions

RRG, FJLG served as the principal investigators and contributed to study design, data collection, and manuscript preparation. LEMG, ELO, JCLA contributed to study design, data collection and manuscript preparation. MRTS contributed to de acylcarnitine measures, participated in statistical analysis and manuscript preparation. JGGG contributed to data collection and manuscript preparation, LGMA participated in statistical analysis and manuscript preparation. BSG, JZVP contributed to the coordination and helped draft the manuscript. All authors read and approved the final manuscript.
